# Association of lipid-lowering drug targets with risk of cutaneous melanoma: a mendelian randomization study

**DOI:** 10.1186/s12885-024-12366-8

**Published:** 2024-05-17

**Authors:** Lusheng Miao, Taosheng Miao, Ying Zhang, Jin Hao

**Affiliations:** 1 Department of Dermatology, Guangdong Provincial Key Laboratory of Major Obstetric Diseases, Guangdong Provincial Clinical Research Center for Obstetrics and Gynecology, The Third Affiliated Hospital of Guangzhou Medical University, Guangzhou, China; 2 Guangzhou University of Chinese Medicine, Guangzhou, China

**Keywords:** Cutaneous melanoma, Lipid-lowering drugs, Proprotein convertase subtilisin/Kexin type 9(PCSK9), Mendelian randomization

## Abstract

**Background:**

Melanoma proliferation is partly attributed to dysregulated lipid metabolism. The effectiveness of lipid-lowering drugs in combating cutaneous melanoma (CM) is a subject of ongoing debate in both in vitro and clinical studies.

**Method:**

This study aims to evaluate the causal relationship between various lipid-lowering drug targets, namely 3-hydroxy-3-methylglutaryl-coenzyme A reductase (HMGCR, targeted by statins), Proprotein convertase subtilisin/kexin type 9 (PCSK9, targeted by alirocumab and evolocumab), and Niemann-Pick C1-like 1 (NPC1L1, targeted by ezetimibe), and the outcomes of cutaneous melanoma. To mimic the effects of lipid-lowering drugs, we utilized two genetic tools: analysis of polymorphisms affecting the expression levels of drug target genes, and genetic variations linked to low-density lipoprotein cholesterol levels and drug target genes. These variations were sourced from genome-wide association studies (GWAS). We applied Summary-data-based Mendelian Randomization (SMR) and Inverse Variance Weighted Mendelian Randomization (IVW-MR) to gauge the effectiveness of these drugs.

**Results:**

Our findings, with SMR results showing an odds ratio (OR) of 1.44 (95% CI: 1.08–1.92; *P* = 0.011) and IVW-MR results indicating an OR of 1.56 (95% CI: 1.10–2.23; *P* = 0.013), demonstrate a positive correlation between PCSK9 expression and increased risk of CM. However, no such correlations were observed in other analyses.

**Conclusion:**

The study concludes that PCSK9 plays a significant role in the development of CM, and its inhibition is linked to a reduced risk of the disease.

**Supplementary Information:**

The online version contains supplementary material available at 10.1186/s12885-024-12366-8.

## Introduction

Cutaneous melanoma (CM), a life-threatening skin cancer, is responsible for 77% of all skin cancer-related deaths [1, 2]. The incidence of CM is on the rise, increasing at an approximate rate of 3% annually [2]. Characterized by its high invasiveness, melanoma in some patients is resistant to most treatment methods due to specific genetic mutations. This resistance, coupled with dosage limitations, underscores the necessity for combination therapy [3–5]. Beyond the primary prevention of ultraviolet radiation exposure, the early chemoprevention of melanoma is also imperative given its challenging treatment and poor prognosis [2].

Most cancers, including melanoma, rely on lipids and cholesterol for their energy needs [6]. Lipid-lowering drugs, particularly statins, are promising candidates for chemoprevention. Their widespread use and established long-term safety make them suitable for this role. As commonly used drugs with well-defined targets, repurposing them is more efficient and cost-effective than developing new medications. Additionally, when combined with lipid abnormalities, they can provide personalized treatment. This study focuses on FDA-approved lipid-lowering drugs, specifically namely 3-hydroxy-3-methylglutaryl-coenzyme A reductase (HMGCR) inhibitors, Proprotein convertase subtilisin/kexin type 9 (PCSK9) inhibitors, and NPC1-like Niemann-Pick C1–like 1 (NPC1L1) inhibitors, as potential melanoma chemoprevention agents.

The effectiveness of lipid-lowering drugs in treating CM remains controversial. Some studies, such as one showing an association between lovastatin use and reduced melanoma incidence (OR, 0.52; 95% CI: 0.27–0.99, *p* = 0.04), suggest potential benefits [7]. In vitro research has demonstrated that statins can inhibit melanoma metastasis and augment treatment in BRAF inhibitor-resistant melanomas when used with other drugs [3]. However, a substantial clinical study with 1318 cases and 6786 controls (OR, 0.98; 95% CI: 0.78–1.20) found no significant association between statin use and CM risk [2]. PCSK9’s role in melanoma progression, through its impact on lipid metabolism and the immune system, has been highlighted in vitro studies [5, 8]. Research on NPC1L1 inhibitors in this context is still in its infancy.

Mendelian Randomization (MR), leveraging the principle of random allocation at conception, can emulate Randomised Controlled Trials by eliminating confounding biases and reverse causality in the research process [9]. For our study, we focused on HMGCR, PCSK9, and NPC1L1 as drug targets in the Mendelian Randomization analysis.

## Methods

Our MR utilizes publicly aggregated data from expression quantitative trait loci (eQTL) studies and genome-wide association studies (GWAS), as detailed in Additional file 1. This study employs both SMR and MR methods. SMR demonstrates significantly higher power compared to two independent large-sample MR analyses when potential non-genetic confounders are present. Unlike two-sample MR testing methods, SMR utilizes the HEIDI detection method to distinguish pleiotropy from linkage by incorporating multiple SNPs within the cis-eQTL region, effectively eliminating their interference [10]. The mutual validation between these two methods enhances the reliability of the results. All original studies involved have received ethical approval.

### Genetic variants for lipid-lowering drugs

In the Summary-data-based Mendelian Randomization (SMR) analysis, eQTLs related to the drug target genes HMGCR, PCSK9, and NPC1L1 were used as surrogate markers for exposure to lipid-lowering drugs. The eQTLs data for HMGCR originated from the eQTLGen Consortium (https://www.eqtlgen.org/), whereas PCSK9 and NPC1L1 data were sourced from adipose tissue in the GTEx database (https://gtexportal.org/). We identified significant common single nucleotide polymorphisms (SNPs, with a minor allele frequency [MAF] > 1%) associated with the expression levels of these genes in specific tissues. Specifically, HMGCR and PCSK9 in blood, and NPC1L1 in subcutaneous adipose tissue were examined. For genetic tool construction, we used cis-eQTLs located within a 1 Mb range of the coding genes. These were selected based on a significance level defined by p-values below 5.0 × 10^− 8^.

For the Inverse Variance Weighted Mendelian Randomization (IVW-MR) analysis, we used low-density lipoprotein cholesterol data from 440,546 participants of both genders in the UK Biobank, accessible via the IEU website (https://gwas.mrcieu.ac.uk) [11]. To accurately represent exposure to lipid-lowering medications, SNPs within a 100 kb range of each drug target gene were selected based on their significant genome-wide associations with LDL cholesterol levels (MAF > 1%, p-value < 5.0 × 10^− 8^). Additionally, to ensure the robustness of each drug as an instrument, these SNPs were chosen for their minimal linkage disequilibrium with one another (r² < 0.30), enhancing the integrity of the analysis [12]. The targets for this analysis were HMGCR, PCSK9, and NPC1L1. Examples of drugs targeting these genes include lovastatin and simvastatin for HMGCR, alirocumab and evolocumab for PCSK9, and ezetimibe for NPC1L1 inhibitors.

### Genetic variants for cutaneous melanoma

For our study on CM, we sourced genetic associations from the most recent FinnGen study (Release 10) [13]. The FinnGen study is a large-scale genomics initiative that has analyzed over 500,000 Finnish biobank samples and correlated genetic variation with health data to understand disease mechanisms and predispositions. The project is a collaboration between research organisations and biobanks within Finland and international industry partners. Our study encompassed a cohort of 5,621 patients with CM and 252,323 controls. Cases of CM were identified using the International Classification of Diseases, Tenth Revision (ICD-10) code L40.

### Genetic variants for coronary heart disease

To ascertain the appropriateness of gene variants as targets for lipid-lowering drugs, we conducted a positive control analysis focusing on coronary heart disease (CHD). The data for this analysis was derived from the CARDIoGRAMplusC4D consortium, which included a substantial sample size of 60,801 CHD cases and 123,504 controls [14].

### Statistical analysis

#### SMR and sensitivity analyses

Given that our study investigated three related drug targets, we employed a Bonferroni-corrected P-value threshold of less than 0.017 (0.05/3) to identify strong evidence of association [15].

To assess the link between the expression of lipid-lowering drug targets and cutaneous melanoma, we initially applied SMR, which incorporated summary data from eQTLs and GWAS studies (Additional file 1). A HEIDI test yielding a p-value less than 0.01 indicated the presence of pleiotropy, suggesting that the associations observed might be attributable to linkage disequilibrium [12] (Additional file 2).

#### MR and sensitivity analyses

In the IVW-MR analysis [16], we focused on genetic variants related to LDL cholesterol levels as instrumental variables. We included only those SNPs with an F-statistic greater than 10, ensuring a robust correlation between the instrument and the exposure (Additional file 3) [17]. To verify that our selected drug targets did not influence melanoma outcomes through other risk factors, we utilised the PhenoScanner [18, 19], a genotype-phenotype database, to investigate associations between the variants targeting each drug and other traits that could signify pleiotropic pathways. Owing to the established correlation between body weight, diabetes, and cutaneous melanoma, we excluded SNPs associated with body weight and diabetes (*p* < 1 × 10^− 5^) from the HMGCR and PCSK9 analyses [20–24]. Various analytical methods, including IVW, the weighted median approach [25], and MR Egger [26], were employed. The fixed-effect model of IVW was primarily utilised for evaluations, as it provides reliable causal estimates even amid heterogeneity [27]. The weighted median estimator offers a consistent causal assessment when over half of the instrumental variables are deemed valid.

To thoroughly evaluate heterogeneity and pleiotropy and ensure the robustness of our findings — particularly that the outcomes are not influenced by other risk factors linked to the exposure — we employed Cochran’s Q statistic and the MR-Egger test (intercept) [28]. When significant heterogeneity was detected (*P* < 0.05), the multiplicative random effects IVW method was utilised. In instances of observed horizontal pleiotropy, the MR-Egger test (with an intercept-related P-value < 0.05) was adopted as our primary analytical approach [26]. Additionally, the MR-PRESSO was implemented for further pleiotropy correction [29]. The methodology for the positive control analysis CHD was conducted in the same manner as previously described (Additional file 2).

In this study, software version 1.03 was used for SMR analysis (details available at: https://cnsgenomics.com/software/smr/#Overview). Additionally, two-sample data analysis was conducted using R version 4.2.2 with the TwoSampleMR package.

## Results

**Fig. 1:**
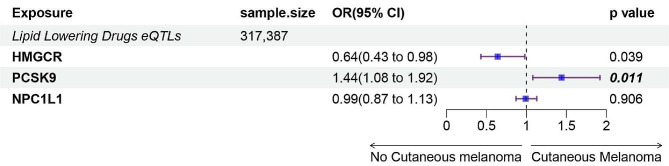
Summary data based Mendelian randomization (SMR) association between expression of gene. eQTL, expression quantitative trait loci; HMGCR, PCSK9, or NPC1L1 and Cutaneous Melanoma outcomes. In this forest plot, the line’s beginning points represent the confidence interval’s lower and upper limits, respectively. The central blue square denotes the odds ratio (OR). An extended purple line segment crossing the threshold of 1 (OR > 1) indicates a heightened risk of cutaneous melanoma. IVW, inverse-variance weighted; WM, Weighted median; HMGCR,3-Hydroxy-3-Methylglutaryl-CoA Reductase; NPC1L1, NPC1 Like Intracellular Cholesterol Transporter 1; PCSK9, Proprotein convertase subtilisin/kexin type 9

In our Summary-data-based Mendelian Randomization (SMR) analysis, a significant association was identified between the PCSK9 drug target and the risk of cutaneous melanoma (OR, 1.44; 95% CI: 1.08–1.92; *p* = 0.011) as shown in Fig. 1. This finding suggests that inhibitors of PCSK9 might have the potential to reduce the risk of this skin cancer. However, after applying the Bonferroni correction, no significant associations were observed between either HMGCR (*p* = 0.039) or NPC1L1 (*p* = 0.906) and cutaneous melanoma. The HEIDI Test, applied to evaluate pleiotropy, indicated no pleiotropy in the analyses of these three drug targets (Additional file 4).

**Fig. 2:**
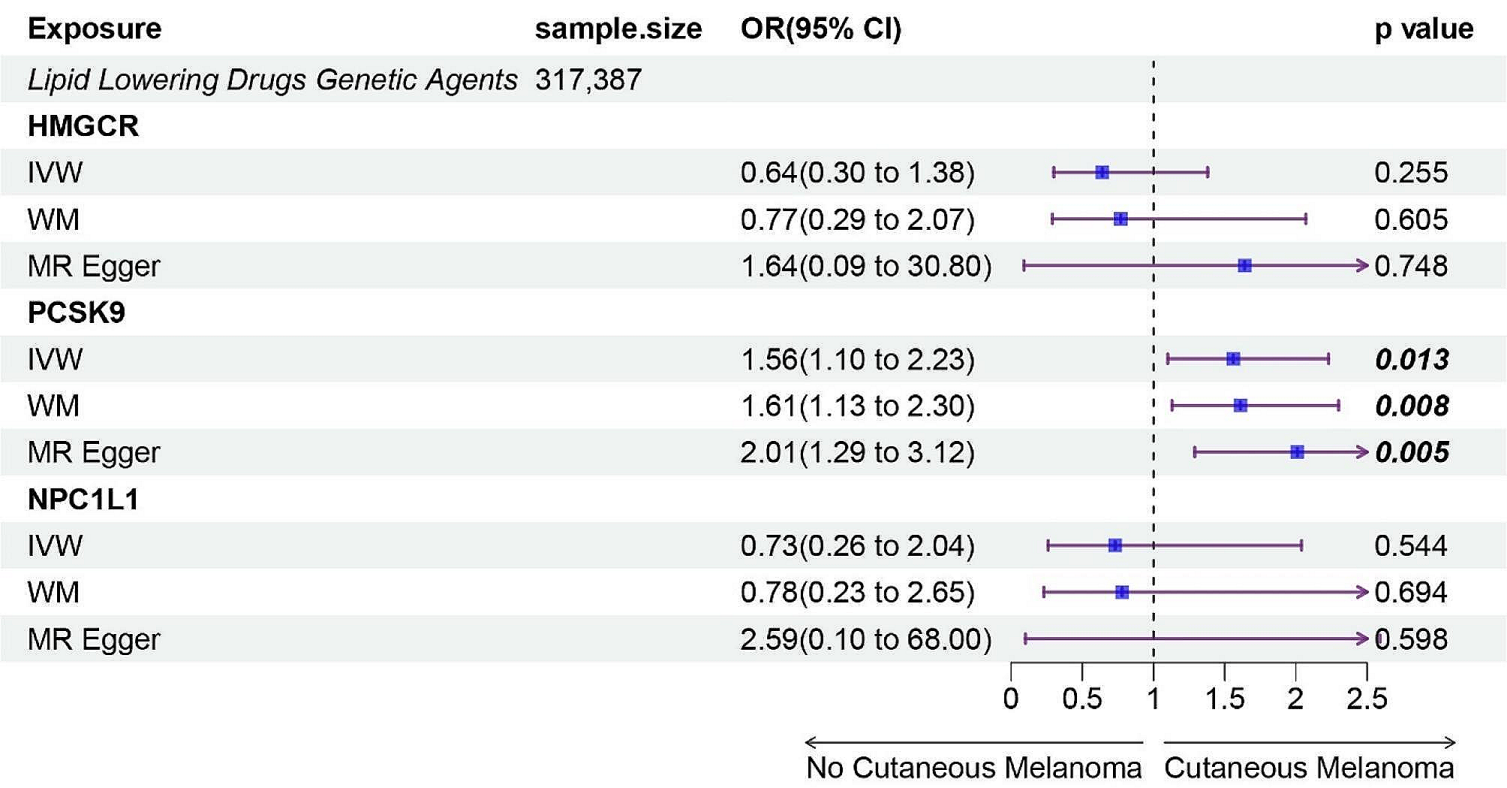
Inverse-variance-weighted Mendelian randomization (IVW-MR) association between low-density lipoprotein (LDL) cholesterol mediated by gene HMGCR, PCSK9, or NPC1L1 and cutaneous melanoma outcomes. IVW- MR method was used to assess the association. The central blue square denotes the odds ratio (OR). An extended purple line segment crossing the threshold of 1 (OR > 1) indicates a heightened risk of cutaneous melanoma; MR, Mendelian randomization; HMGCR,3-Hydroxy-3-Methylglutaryl-CoA Reductase; NPC1L1, NPC1 Like Intracellular Cholesterol Transporter 1; PCSK9, Proprotein convertase subtilisin/kexin type 9

In the IVW MR analysis, we used 11 HMGCR, 27 PCSK9, and 6 NPC1L1 instrumental variables in our final assessment (Additional file 5–6). The analysis revealed that the PCSK9 drug target is associated with an increased risk of cutaneous melanoma (OR, 1.56; 95% CI: 1.10–2.23; *p* = 0.013), as illustrated in Fig. 2. No significant associations were found with the HMGCR drug target and NPC1L1 drug target. Notably, heterogeneity was detected in the PCSK9 analysis, which was adjusted using the multiplicative random effects IVW method [27]. Cochran’s Q test showed no evidence of heterogeneity in other reported outcomes (all *p* > 0.05). Additionally, no significant overall pleiotropy was detected as per the MR-Egger regression intercept term and MR PRESSO (Additional file 7).

**Fig. 3:**
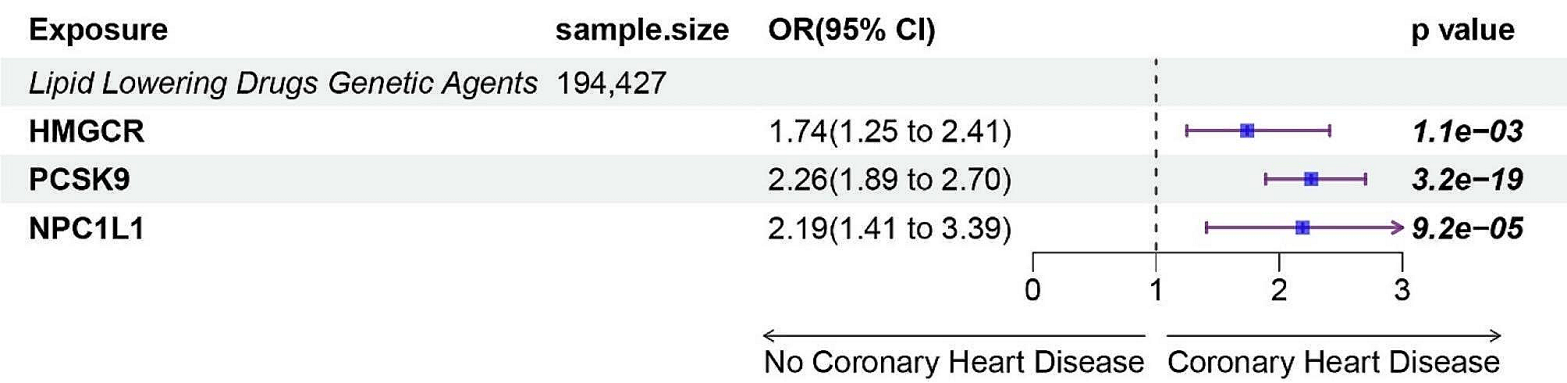
Inverse-variance-weighted Mendelian randomization (IVW-MR) association between low-density lipoprotein (LDL) cholesterol mediated by gene HMGCR, PCSK9, or NPC1L1 and coronary heart disease outcomes. IVW- MR method was used to assess the association. The central blue square denotes the odds ratio (OR). An extended purple line segment crossing the threshold of 1 (OR > 1) indicates a heightened risk of cutaneous melanoma; MR, Mendelian randomization; HMGCR,3-Hydroxy-3-Methylglutaryl-CoA Reductase; NPC1L1, NPC1 Like Intracellular Cholesterol Transporter 1; PCSK9, Proprotein convertase subtilisin/kexin type 9

The positive control analysis, involving instrumental variables from IVW MR, demonstrated that all three drug target proxies were associated with cardiovascular diseases (Fig. 3, Additional file 8). All conducted sensitivity analyses indicated the absence of both heterogeneity and horizontal pleiotropy (Additional file 9), reinforcing the appropriateness of these genetic variations as drug targets.

## Discussion

The main discovery of this study is that PCSK9 may serve as a potential therapeutic target for skin melanoma. Our findings indicate that existing PCSK9 inhibitors, such as alirocumab and evolocumab, can influence melanoma by targeting LDL-related sites within the gene, thereby supporting the repurposing of these drugs. Previous research has shown that inhibiting PCSK9 can be effective in various cancers, including lung, colorectal, breast cancers [30–32], suggesting its broad potential in oncology. Consequently, targeting PCSK9 could be promising for the preventive treatment of melanoma.

The protease PCSK9 is integral to cholesterol balance regulation, mainly through its interaction and subsequent degradation of low-density lipoprotein receptors (LDLR) [5]. The accelerated growth of melanoma and changes in its immune characteristics are partly due to lipid metabolism disorders [8]. Cancer cells increase their lipid biosynthesis by absorbing exogenous fatty acids, thus facilitating rapid growth. A notable role of PCSK9 is the modulation of MHC (major histocompatibility complex) I expression on cancer cell surfaces, aiding melanoma cells in evading immune detection [5].

Current studies also highlight the potential of curcumin in inhibiting PCSK9’s effect on cutaneous melanoma by regulating oxidative stress-related signaling pathways [33]. PCSK9’s involvement in the development and metastasis of lung and liver melanomas, through LDLR or other mechanisms [34, 35], underscores the potential of PCSK9 inhibitors in reducing melanoma risk and improving prognosis.

Regarding lipid-lowering drugs and melanoma, much focus has been on HMGCR inhibitors, commonly known as statins. The role of statins in reducing melanoma risk and improving prognosis has been widely debated. While some Mendelian randomization research suggests that statins could lower the risk of skin malignant melanoma [1], our extensive study using two analytical methods found no significant association, aligning with previous clinical studies [2, 36, 37]. In line with our findings, related basic research indicates that while statins may not alter the incidence of melanoma, they could potentially influence its growth, metastasis, and other prognostic factors—areas that warrant further exploration [2, 38].

As for the NPC1L1 and its association with cutaneous melanoma, research is limited. Our study indicates no significant correlation with melanoma incidence, necessitating further in vitro and clinical studies for more conclusive evidence.

### Limitation

Our Mendelian study has certain limitations. eQTLs are categorized into cis-QTLs and trans-QTLs. Cis-eQTLs, located within the genomic region of the gene itself, suggest that variations in the gene itself may influence mRNA level changes. In contrast, a trans-eQTL, located in a different genomic region, indicates that variations in other genes control mRNA level differences in the target gene. In this study, we focused solely on cis-eQTLs and did not consider the potential effects of trans-eQTLs, which may introduce some bias into our results.

## Conclusion

This MR study indicates a potential causal connection between PCSK9 and heightened skin melanoma risk, with no association between HMGCR (target of statin drugs) and melanoma onset.

### Electronic supplementary material

Below is the link to the electronic supplementary material.

Supplementary Material 1

**Additional file 2 Overview of the study design**. eQTLs, expression quantitative trait loci; GWAS, genome-wide association study; LDL-C, low-density lipoprotein cholesterol; HMGCR, 3-Hydroxy-3-Methylglutaryl-CoA Reductase; PCSK9, Proprotein convertase subtilisin/kexin type 9; NPC1L1, NPC1 Like Intracellular Cholesterol Transporter 1; MR, Mendelian randomization; SMR, Summary data-based Mendelian randomization; HEIDI, heterogeneity in dependent instruments; IVW, inverse-variance weighted; WM, Weighted median

Supplementary Material 3

Supplementary Material 4

**Additional file 5 The flowchart of the selection of instrumental variables (IVs) in IVW-MR.** LDL-C, low-density lipoprotein cholesterol; GWAS, genome-wide association study; HMGCR, 3-Hydroxy-3-Methylglutaryl-CoA Reductase; PCSK9, Proprotein convertase subtilisin/kexin type 9; NPC1L1, NPC1 Like Intracellular Cholesterol Transporter 1; CM, cutaneous melanoma; MR, Mendelian randomization; CHD, coronary heart disease.

Supplementary Material 6

Supplementary Material 7

Supplementary Material 8

Supplementary Material 9

## Data Availability

All data generated or analysed during this study are included in this published article [Additional file 1].
