# Supplementary figures and images for: Association of lipid-lowering drug targets with risk of cutaneous melanoma: a mendelian randomization study
Source: BMC Cancer. 2024 May 17;24:602. doi: 10.1186/s12885-024-12366-8 (PMC11102253; doi:10.1186/s12885-024-12366-8)

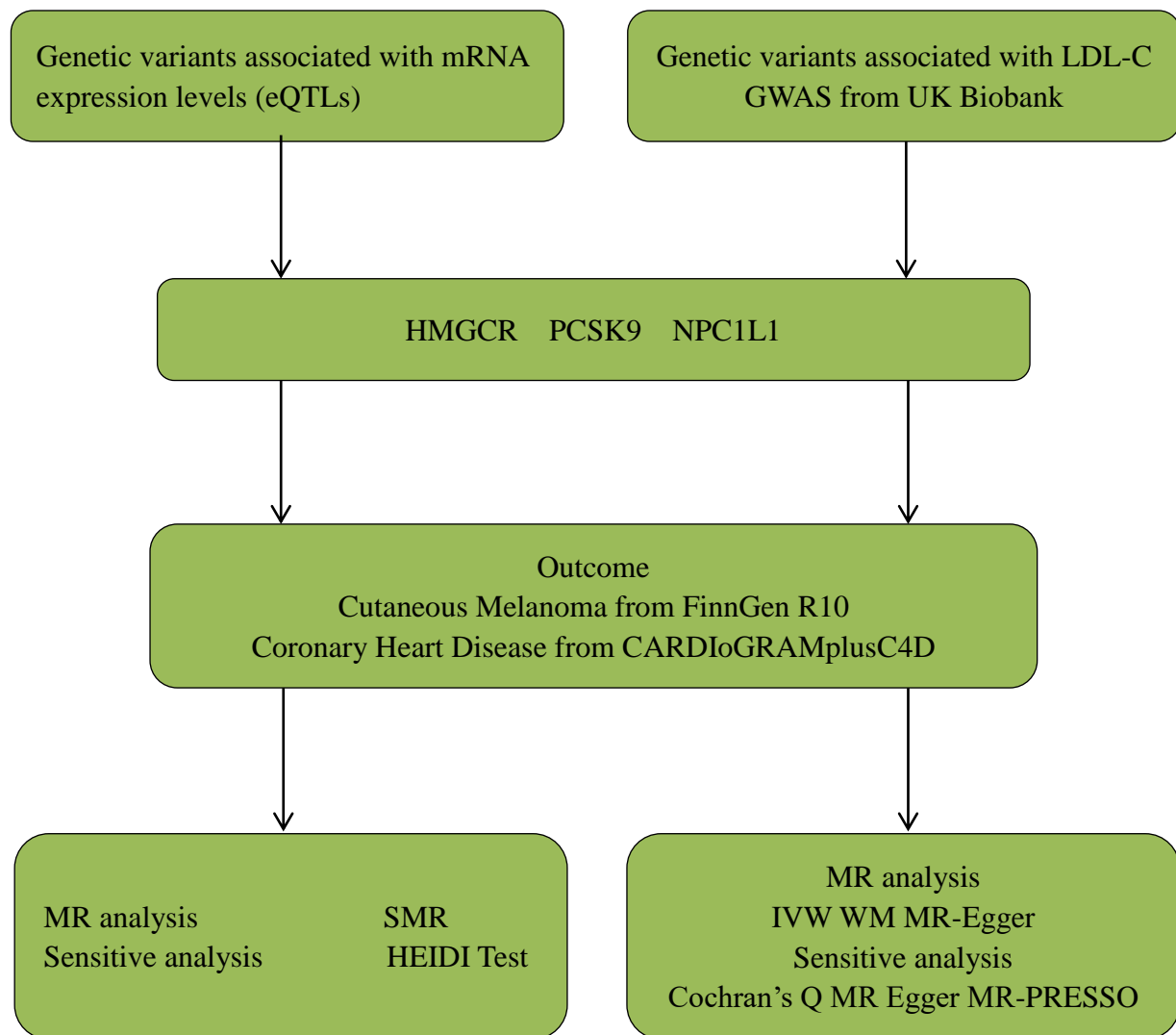

Supplement: Supplementary file 2 — Additional file 2 Overview of the study design. eQTLs, expression quantitative trait loci; GWAS, genome-wide association study; LDL-C, low-density lipoprotein cholesterol; HMGCR, 3-Hydroxy-3-Methylglutaryl-CoA Reductase; PCSK9, Proprotein convertase subtilisin/kexin type 9; NPC1L1, NPC1 Like Intracellular Cholesterol Transporter 1; MR, Mendelian randomization; SMR, Summary data-based Mendelian randomization; HEIDI, heterogeneity in dependent instruments; IVW, inverse-variance weighted; WM, Weighted median [file 12885_2024_12366_MOESM2_ESM.pdf]

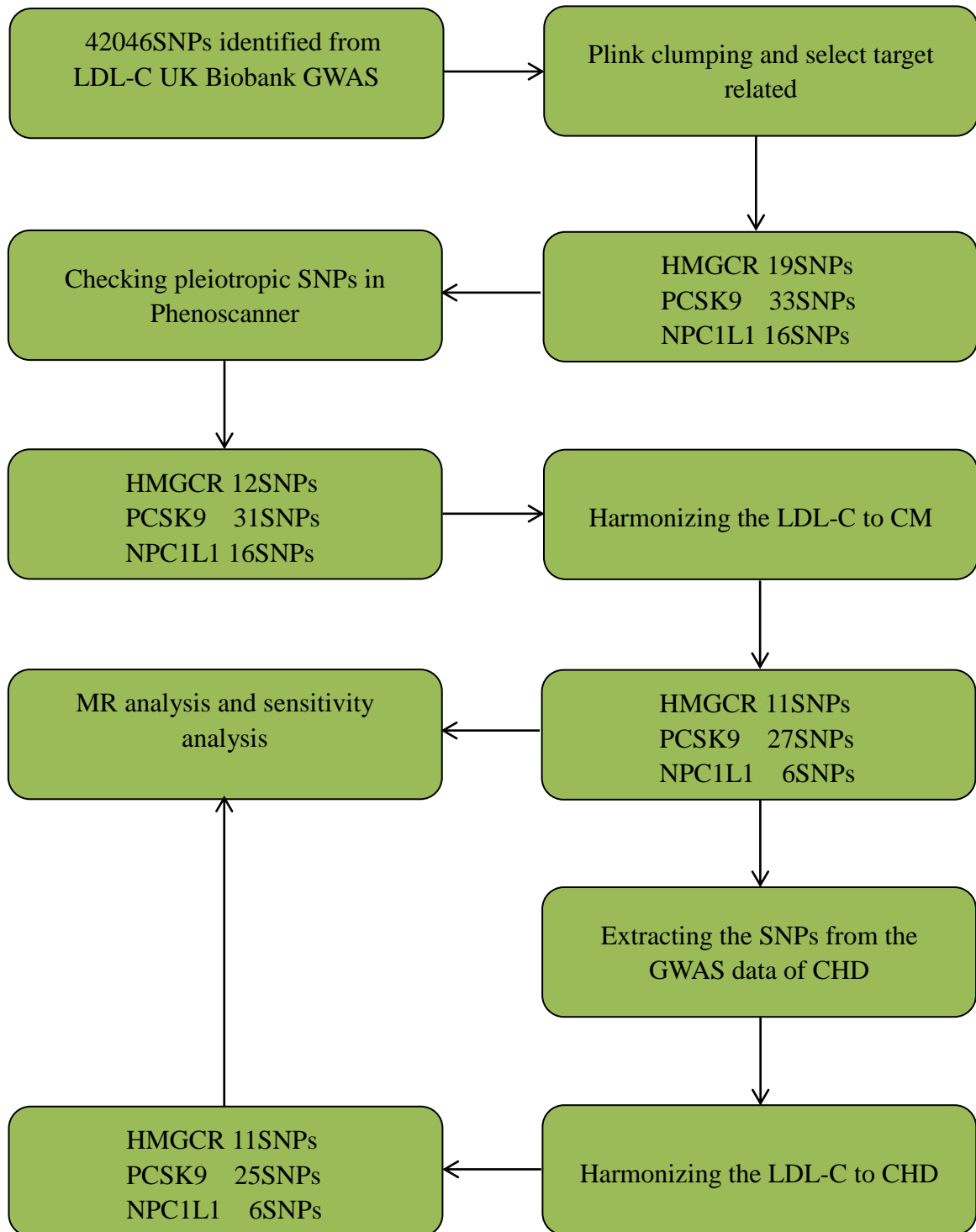

Supplement: Supplementary file 5 — Additional file 5 The flowchart of the selection of instrumental variables (IVs) in IVW-MR. LDL-C, low-density lipoprotein cholesterol; GWAS, genome-wide association study; HMGCR, 3-Hydroxy-3-Methylglutaryl-CoA Reductase; PCSK9, Proprotein convertase subtilisin/kexin type 9; NPC1L1, NPC1 Like Intracellular Cholesterol Transporter 1; CM, cutaneous melanoma; MR, Mendelian randomization; CHD, coronary heart disease. [file 12885_2024_12366_MOESM5_ESM.pdf]
